# sEMG feature evaluation for identification of elbow angle resolution in graded arm movement

**DOI:** 10.1186/1475-925X-13-155

**Published:** 2014-11-25

**Authors:** Maria Claudia F Castro, Esther L Colombini, Plinio T Aquino Junior, Sridhar P Arjunan, Dinesh K Kumar

**Affiliations:** Electrical Engineering Department, Centro Universitário da FEI, Av. Humberto de A. C. Branco, 3.972, São Bernardo do Campo, SP 09850-901 Brazil; Computer Science Department, Centro Universitário da FEI, Av. Humberto de A. C. Branco, 3.972, São Bernardo do Campo, SP 09850-901 Brazil; Biosignal Lab., School of Electrical and Computer Engineering, RMIT University, GPO Box 2476, Melbourne, VIC 3001 Australia

**Keywords:** EMG signal, Pattern recognition, Feature extraction, Angular position, Arm flexion/extension

## Abstract

Automatic and accurate identification of elbow angle from surface electromyogram (sEMG) is essential for myoelectric controlled upper limb exoskeleton systems. This requires appropriate selection of sEMG features, and identifying the limitations of such a system.

This study has demonstrated that it is possible to identify three discrete positions of the elbow; full extension, right angle, and mid-way point, with window size of only 200 milliseconds. It was seen that while most features were suitable for this purpose, Power Spectral Density Averages (PSD-Av) performed best. The system correctly classified the sEMG against the elbow angle for 100% cases when only two discrete positions (full extension and elbow at right angle) were considered, while correct classification was 89% when there were three discrete positions. However, sEMG was unable to accurately determine the elbow position when five discrete angles were considered. It was also observed that there was no difference for extension or flexion phases.

## Background

Exoskeleton systems of the arm have number of applications such as support for the elderly, defense personnel, and people with skeletal injuries [1–3]. For effective application of these devices, the user should be able to control them naturally and intuitively. While there are number of options for commanding such systems such as the use of mechanical sensors, brain computer interface and use of surface electromyogram (sEMG) of the associated muscles, sEMG provides a natural and intuitive interface for the user [3–9]. This can also offer the user with proportional control where the exoskeleton device can follow the body movement. However, the difficulty with such sEMG based controlling strategies is the poor sensitivity and specificity, leading to poor reliability.

The angle of the elbow is an important command of the exoskeleton of the upper limb. To obtain this from sEMG recording requires the appropriate selection of sEMG features which then have to be classified to identify the elbow angle. Different researchers have used different features [10–14]. However, none of the researchers have performed a comparative between all of these features.

Proportional control requires the system to identify the position of the body based on the sEMG of the effective muscles. While some simple systems provide binary resolutions such as flexion and extension commands, this does not offer proportional control, and is not intuitive [15–18]. There is the need for higher resolution where the user is able to give finer commands to the exoskeleton device for performing the upper limb actions. Higher resolution requires the classification system to have larger number of classes. However, there is a tradeoff between the number of classes and the system accuracy and reliability, and there is the need for determining the relationship between the number of classes and the system accuracy.

The aim of this research was to determine the relationship between the classification system sensitivity, specificity and accuracy for different resolutions of the elbow angles (number of classes or number of arm positions), and determine the feasibility of high resolution identification of the elbow angle. A comparison was performed between the commonly used features of sEMG to select the most suitable feature set for the proposed classification system. The accuracy, sensitivity and specificity of each of these features in the recognition of the elbow angle were obtained. The relationship between resolution (number of classes) and the accuracy, sensitivity and specificity of recognition of the position of the arm was studied.

## Methods

The experimental protocol was approved by the Research Ethics Committee from São Judas University, São Paulo, Brazil, by letter; COEP-USJT-No.076-2010, and in accordance with the Helsinki accord (modified 2004). Seven able-bodied volunteer subjects (4 men and 3 women), average age 34.6, participated in the experiment. They were verbally and in writing explained the purpose of the experiments and the experimental protocol, and experiments were performed after obtaining their verbal and written informed consent. Before recording the data, the participants were allowed sufficient time to familiarize themselves with the equipment and the protocol. Multiple trial runs were performed till the volunteers were comfortable with the experiment.

### Experiments

#### Equipment

**Figure 1:**
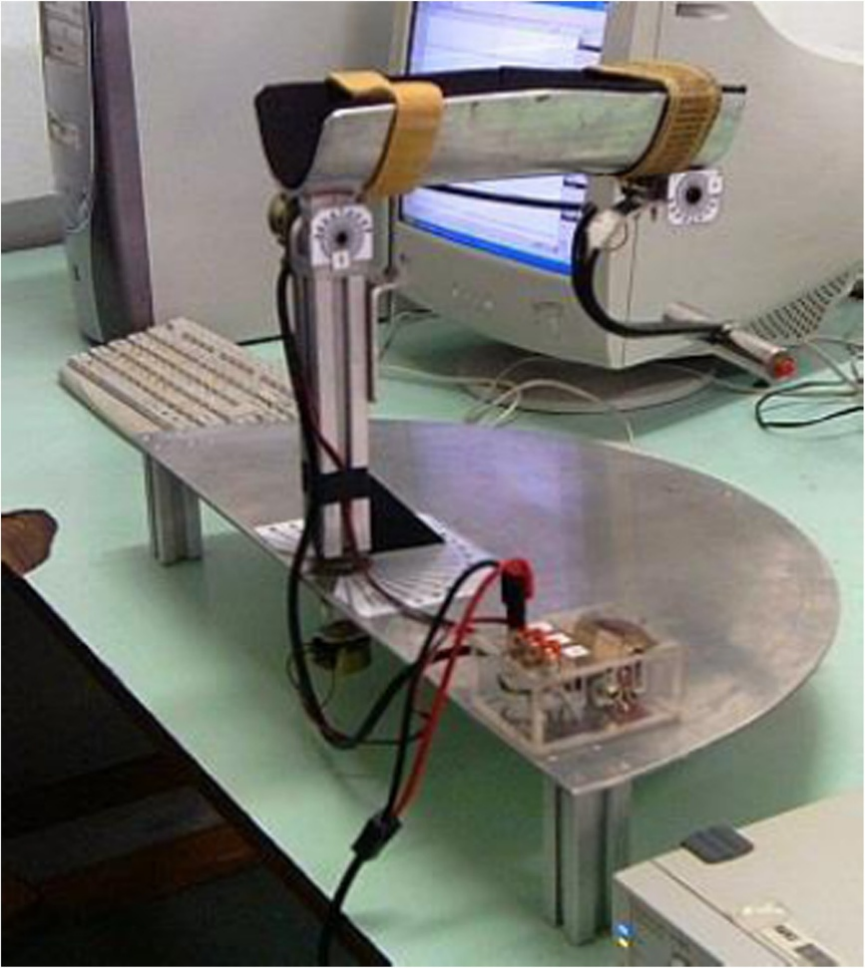
**Custom made elbow angle monitoring device.**

A custom-made elbow angle monitoring device (Figure 1) was used to monitor elbow angular position. This device restricts the movement of the arm at the elbow in the horizontal plane and is fixed at the height of shoulder of the subject. A goniometer records the angle between the upper arm and the forearm at the elbow. The users were given visual feedback of the elbow angle on the screen throughout the experiment.

Two channel sEMG signals were recorded using Powerlab (AdInstruments), using disposable pre-gelled bipolar surface electrodes (Noraxon). The ground electrode was placed on the acromion point. The electrodes, for recording the biceps sEMG, were placed above the motor point of the short head, on the line between the acromion and the fossa cubit, at 1/3 from the fossa cubit. And the electrodes, for the lateral head of the triceps, were placed on the middle of the line between the posterior crista of the acromion and the olecranon at two finger widths lateral to the line. The inter-electrode was maintained at 20 mm (center to center). The signal was sampled at 1000 Hz/channel and filtered using eighth-order, switched-capacitance, Bessel type filter, range 20–500 Hz and notch at 60 Hz.

#### Experimental protocol

During the experiments, the angle of the elbow along with the sEMG from the biceps brachii and triceps brachii was recorded. The participants were given continuous visual feedback of the angle of the elbow.

**Figure 2:**
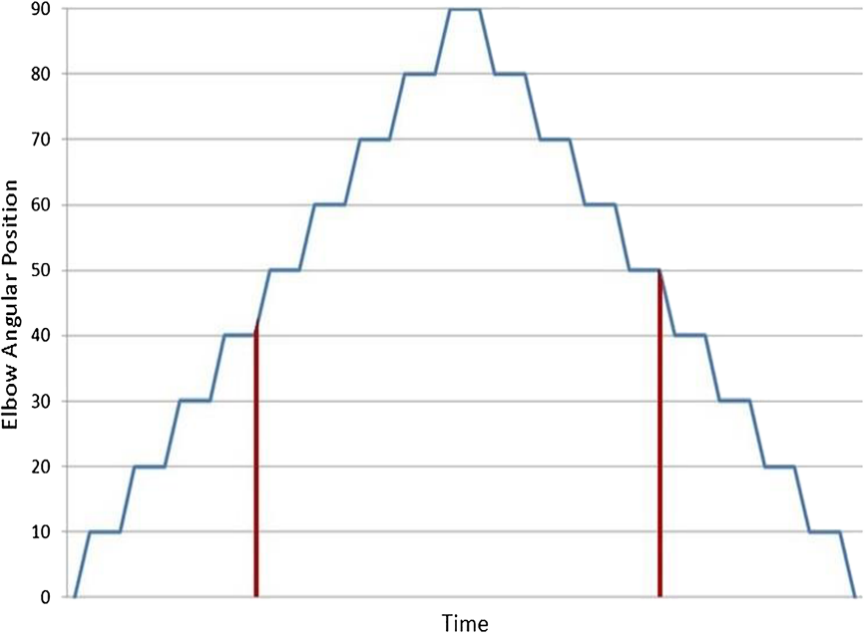
**Graded movement for arm flexion/** **extension showing elbow angular position as a function of time**.

During the experiment, the participant performed graded flexion/extension movements with 10*°* shifts every 3 s, going from full extension position (0*°*) to 90*°* of flexion and returning to the full extension position in the same way (Figure 2). The participants were provided with audio cues for timing the movement. This procedure was repeated 3 times for each volunteer, with 5 minutes rest period between experiments to ensure there was no fatigue.

#### Signal processing

There are two possible techniques for determining the position of the arm from sEMG recordings of the biceps and triceps muscles; dynamic or static. The dynamic relates to the sEMG recorded when the arm is in motion and the muscle is producing the motion, while the static is when the arm is not moving and the muscle activity is isometric. In this situation, contractions above certain threshold are usually used, being stronger than those used in dynamic movement without load [5, 8, 9].

#### Segmentation

Good classification of the signal requires high signal to noise ratio. While isometric contraction during relaxed, maintained position of the arm has very low muscle activity because of which the signal to noise ratio becomes very poor, sEMG during the movement is significantly higher, with higher signal to noise ratio. Thus, dynamic contraction phase during arm movement was selected for the purpose of signal analysis.

Researchers have identified delays need to be less than 250 ms for user satisfaction. Analysis of the signal also showed that first 200 ms of each step movement [10] is consistent and hence was considered for analysis. The signal was segmented; the 200 ms at the start of each 10° shift movement was selected forming one of the data vectors in each data class, and is indicated by two examples shown by the red regions in Figure 2. The signal was normalized based on the maximum value in this range. It was then labeled based on the angle of the elbow such as B_f10_ being the 200 ms segment of sEMG of the biceps obtained at the completion of the 10° flexion. Experimental class set ups were defined in Table 1.

**Table 1:** **Angular interval variation for each class set up**, **for each movement phase**

Number of classes	Angular variation during	Angular variation during
	Flexion phase	Extension phase
**2**-**class set up**	0*°* − 10*°*; 80*°* − 90*°*	90*°* − 80*°*; 10*°* − 0*°*
**3**-**class set up**	0*°* − 10*°*; 40*°* − 50*°*; 80*°* − 90*°*	90*°* − 80*°*; 50*°* − 40*°*; 10*°* − 0*°*
**5**-**class set up**	0*°* − 10*°*; 20*°* − 30*°*; 40*°* − 50*°*; 60*°* − 70*°*; 80*°* − 90*°*	90*°* − 80*°*; 70*°* − 60*°*; 50*°* − 40*°*; 30*°* − 20*°*; 10*°* − 0*°*

#### Feature extraction

Appropriate section of features to represent the sEMG signal is essential for accurate identification of actions and movements [11, 19]. While researchers have tested the efficacy of number of features, few publications have compared the accuracy, sensitivity and specificity of different features. In this work, 9 commonly reported features were extracted. These are briefly described in Table 2, where, *x*_*k*_ is the *k*th sample of a total of *N*, in the window *i* of a total of *I* number of windows [10, 12–14].

**Table 2:** **Feature definition**

Features	Equations
**Mean Absolute Value**	
**Root Mean Square**	
**Waveform Length**	
**Willison Amplitude**	

**Zero Crossings**	

**Autoregressive Model** **(AR)**	
	In this study, p = 6.
**Power Spectral Density**	
**Power Spectral Density Averages**	
**Power Spectral Density Moments**	
	In this study, *w* = 250 Hz and y = 1,2,3

#### Linear discriminant analysis as classifier

Linear Discriminant Analysis (LDA) is a statistical method based on linear transformations of the data set, projected onto the directions that achieve the best class separability. The goal is to maximize the between-class scatter matrix while minimizing the within-class scatter matrix [20, 21].
1

According to Fisher criterion, the solution for the Equation 1, that defines the projection matrix *W*_*lda*_, can be achieved as a typical problem of eigenvectors, which the solution are the eigenvectors and the eigenvalues of , with at most (*g* − 1) nonzero eigenvalues, where *g* is the number of classes [20, 21].

However, in practical applications, the within-class scatter matrix *S*_*w*_ can be singular. This comes from the fact that, in general, the number of patterns in the training set *N*_*i*_ is much smaller than the dimensionality *d* of the data set [20, 21]. To deal with this singularity problem, one of the methods in the literature, known as Regularized LDA (RLDA), adds a constant α to the diagonal elements of the pooled matrix *S*_*p*_ (defined by Equation 2), where α is known as the regularization parameter. In this work, α ranged from 10^-9^ to 0.9, interval defined in a previous study for class separability purposes [22].
2

The analysis was first done when all the subjects were pooled together during training and testing. However, the results from the leave one out technique were very poor, and this approach was discarded. Subsequently, the training was repeated for each subject individually, and these results have been shown in this paper. This also demonstrates that there is significant variation between subjects, and indicates that it is important for the classifier to be trained for each user.

Each feature (Table 2) applied over the 200 ms segment produced a vector corresponding to each of the two muscles; biceps and triceps. These feature vectors were the input to the LDA. Leave One Out method was used to validate the system, where the data set was divided in groups of 2 samples for each class and each subject for training the LDA, while the remaining sample was used for testing for each subject. This was repeated three times to ensure there was no bias. The average of the three trials was obtained and is indicative of the ability for the system to determine the elbow angle, taking into account the differences between multiple samples and the considered class set ups.

## Results

The average accuracy, sensitivity and specificity achieved by each feature, for each movement phase, and for each resolution (class set up) are shown in Table 3. From this table it is observed that for resolution = 2 classes, PSD has the highest accuracy, sensitivity and specificity for both movement directions (flexion and extension), while ZC has the lowest.

**Table 3:** **Performance metrics (Se** – **sensitivity%**; **Sp** – **specificity%**; **Acc** – **accuracy%)**

		2- class set up			3- class set up			5- class set up		
		Se	Sp	Acc	Se	Sp	Acc	Se	Sp	Acc
**PSD**-**Av**	Flex	95	95	95	89	94	89	64	91	64
	Ext	100	100	100	84	92	84	62	90	62
**PSD**	Flex	100	100	100	89	94	89	48	87	48
	Ext	100	100	100	76	88	76	52	88	52
**MAV**	Flex	81	78	93	79	87	79	48	87	48
	Ext	100	86	98	86	89	86	65	88	65
**WL**	Flex	100	78	95	76	88	76	50	88	50
	Ext	100	86	100	79	90	79	51	88	51
**RMS**	Flex	85	79	88	62	81	78	51	87	51
	Ext	100	86	100	75	87	79	62	88	62
**AR**	Flex	86	90	88	81	90	81	45	86	45
	Ext	100	95	98	73	87	73	55	89	55
**PSD**-**Mo**	Flex	100	88	93	68	84	68	41	85	41
	Ext	100	100	100	63	82	64	38	85	38
**WAMP**	Flex	59	50	64	46	72	46	27	82	27
	Ext	75	73	79	57	76	57	27	82	28
**ZC**	Flex	75	68	81	52	73	52	33	82	33
	Ext	45	45	50	41	67	41	24	81	24

From this table it is also observed that PSD-Av also has high sensitivity, specificity and accuracy for classification of 2 (full extension and 90° flexion) and 3-class (including a half-way position class) set ups. However, for the configuration using 3 classes, during extension phase, the accuracy is 84%, sensitivity = 84%, and specificity = 92%. Such a system may not be suitable for applications where any error in recognizing the command may cause injury to the user.

The results also show that the response of the system to classify 5-class set up decreases, which shows that no sEMG feature of the biceps and triceps is suitable for accurately identifying the elbow angle for exoskeleton control for higher resolutions. The results were also confirmed by the Kappa based comparative statistics [23] to show the interobserver variation and reported in Table 4. Based on the Cohen’s Kappa statistic value from Table 4 and the study by Viera and Garret [23] it is shown that the Kappa value above 0.60 suggests the substantial agreement with the predicted and actual observation.

**Figure 3:**
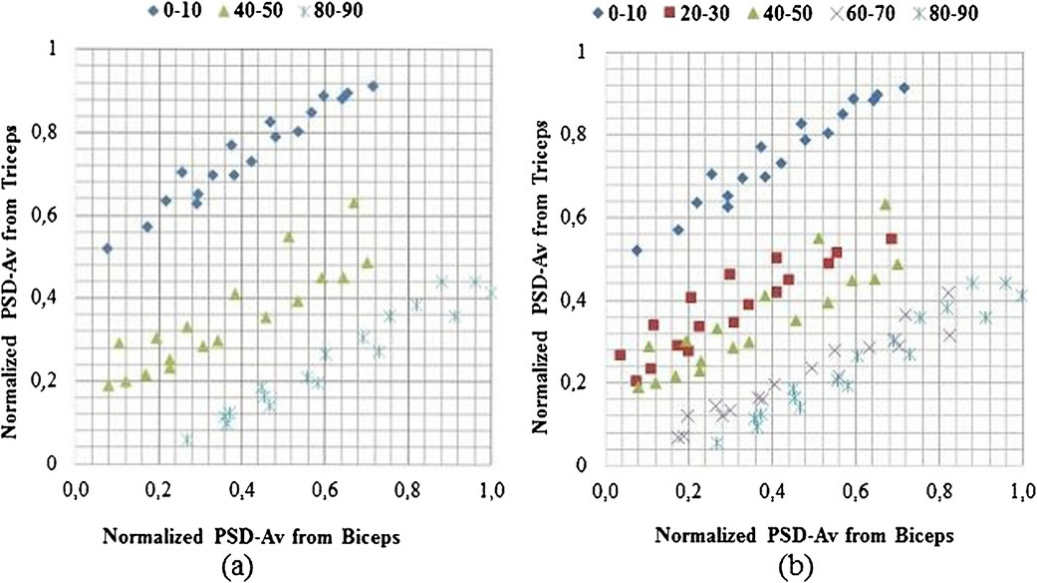
**Example of scatter-** **plots of normalized PSD-** **Av data values during the flexion movement phase for:** **(a) 3-** **class set up and (b) 5-** **class set up.**

The scatter plots of the normalized PSD-Av of the triceps vs biceps are shown in Figure 3. PSD-Av is a feature which consists of 16 parameters for each muscle, and was selected because statistical analysis confirmed it to have the lowest error.

Figure 3(a) is a plot for 3 classes; 0°-10°, 40°-50° and 80°-90° while, Figure 3(b) is a plot for 5 classes; 0°-10°, 20°-30°, 40°-50°, 60°-70° and 80°-90°. From these plots, it is observed that there are significant differences in the 0°-10°, 40° -50° and 80°-90° classes, but there is significant overlap when two additional classes; 20°-30°, and 60°-70° are added. This demonstrates the limitation of such an approach for myoelectric exoskeleton systems.

**Table 4:** **Kappa statistic results**

		Cohen’s Kappa statistics		
		2 class	3 class	5 class
**PSD-Av**	Flex	0.90	0.83	0.55
	Ext	1.00	0.76	0.60
**PSD**	Flex	1.00	0.83	0.35
	Ext	1.00	0.64	0.40
**MAV**	Flex	0.59	0.60	0.37
	Ext	0.85	0.67	0.40
**WL**	Flex	0.74	0.64	0.38
	Ext	0.85	0.69	0.41
**RMS**	Flex	0.73	0.42	0.33
	Ext	0.84	0.62	0.42
**AR6**	Flex	0.76	0.71	0.31
	Ext	0.95	0.60	0.43
**PSD-Mo**	Flex	0.86	0.53	0.26
	Ext	1.00	0.45	0.23
**WAMP**	Flex	0.09	0.17	0.08
	Ext	0.48	0.29	0.08
**ZC**	Flex	0.43	0.19	0.12
	Ext	0.10	0.01	0.05

## Discussion and conclusions

The results show that sEMG system can be used effectively to identify the flexion and extension of the elbow when we consider two state situations; arm at full extension, and arm at 90° flexion. This work has also shown that when the number of classes of classification increased to 3 classes, the system accuracy dropped, with sensitivity = 84% and specificity = 92%. Such a system may be suitable for limited applications due to the relatively low sensitivity, which could cause injury to the user.

When the number of classes was increased to 5-class set up, the error was higher compared with the situation of 2 and 3-class set ups, with sensitivity = 64%, and specificity = 91% in the best case. This indicates that sEMG classification is suitable for the identification of small number of elbow positions but unsuitable for being used for high resolution conditions. Poor sensitivity will frustrate the user and make the system non-functional. One reason for poor sensitivity may be due to the narrow window of 200 ms. However, this is essential because earlier studies have identified that delays greater than 250 ms causes observable delays to the user, and can be the cause of errors. The other reason is the similarities between consecutives positions due to its discretization.

The results also showed that at small number of elbow positions, the performance of most features was similar, but as the number become higher, none of them achieved reasonable results. While relating sEMG with angles in between the extreme flexion and extension did not give good results, however, it should be noted that nil error in detecting full flexion and extension levels is not comparable with relatively higher errors during the in-between steps. But the relatively large error highlights the relatively limited applications of such an approach.

There are other options that may be considered as an alternate to a classification of small number of sEMG channels; high density myoelectric recordings, mechanical sensor system, a hybrid system, or the use of an intelligent myoelectric system where model based approach may be used. While mechanical sensor systems have number of shortcomings, the hybrid system that combines the sEMG with the mechanical sensors may reduce the errors while providing the user with natural and intuitive control. Another approach, where the mechanical sensing may not be possible, is to develop an intelligent system modeling the movement, such as is trained to estimate the speed of the action of the user. In this approach, the system would be trained to estimate the time of the action for an individual and assuming the movement to be continuous. Such a system could provide an alternate for a myoelectric based proportional controller system also known as tracking systems.
